# Micelle and Bilayer Formation of Amphiphilic Janus Particles in a Slit-Pore

**DOI:** 10.3390/ijms13089431

**Published:** 2012-07-26

**Authors:** Gerald Rosenthal, Sabine H. L. Klapp

**Affiliations:** Institute of Theoretical Physics, School II, Technical University Berlin, Sec. EW 7-1, Hardenbergstr. 36, Berlin D-10623, Germany; E-Mail: klapp@physik.tu-berlin.de

**Keywords:** Janus particles, amphiphilic systems, confinement, cluster formation, micelles, icosahedrons, bilayers

## Abstract

We employ molecular dynamics simulations to investigate the self-assembly of amphiphilic Janus particles in a slit-pore consisting of two plane-parallel, soft walls. The Janus particles are modeled as soft spheres with an embedded unit vector pointing from the hydrophobic to the hydrophilic hemisphere. The structure formation is analyzed via cluster size distributions, density and polarization profiles, and in-plane correlation functions. At low temperatures and densities, the dominating structures are spherical micelles, whereas at higher densities we also observe wall-induced bilayer formation. Finally, we compare the MD results with those from a previous density functional study.

## 1. Introduction

The term “Janus”-particles (named after the two-faced roman god) generally refers to particles composed of at least two chemically or physically distinctive surfaces. Significant experimental progress over the last years (see, e.g., [1–6]) nowadays allows us to create a wide class of Janus particles, including particles with “conventional” amphiphilic, but also with electric dipolar or quadrupolar, and even magnetic properties. As a result of this variety, Janus particles have a broad range of technological applications. For example, they are used for the stabilization of emulsions [7,8], but also in water-repellent textiles [9], in electronic paper [10,11] and as microrheological probes [12,13]. From a more fundamental point of view, a very attractive feature of Janus particles is their ability to self-assemble into a variety of structures such as micelles [1,14–16] or chains [17], as well as mesoscale structures such as membranes [15,16,18]. Due to this rich behavior which can be manipulated by external fields [17], Janus particles are considered as well-controllable candidates for the bottom-up design of future materials.

In the present study we focus on amphiphilic spherical Janus particles, where one hemisphere has hydrophilic properties (*i.e*., it attracts water), and the other one is hydrophobic (*i.e*., water-repellent). Our main question is how a suspension of such particles behaves in presence of spatial confinement realized by two planar surfaces. In particular, we are interested in the system’s aggregation properties. To this end we carry out classical equilibrium Molecular Dynamic (MD) simulation of a suitable model system.

We note that there exists quite a number of experimental and theoretical studies on the related problem of the self-assembly of *surfactant molecules* at surfaces (see, e.g., [19,20]), as well as on other interface-dominated problems such as the dispersion of carbon nanotubes in surfactant solutions [21]. For Janus particles, on the other hand, the impact of surfaces is much less understood. In a study by Hirose *et al*., a macroscopic approach (based on Young’s equation) was used to study the wetting properties of Janus systems [22], but not their self-assembly. More recently, we have proposed a density functional theory (DFT) approach [23], where detailed properties such as density and orientation profiles at the surface can be calculated on the basis of the microscopic Hamiltonian. Indeed, to describe the interaction between such particles on an *effective* level (that is, without taking explicitly into account the solvent), various models have been proposed [1,16,18,24]. In [23] we employed a model originally suggested by Tarazona *et al*. [18] to describe surfactant *molecules* on a highly coarse-grained level, that is, by representing the surfactant as a sphere. The orientation of the resulting Janus particles is incorporated by a unit vector pointing from the hydrophobic to the hydrophilic side. While the model is appropriate for DFT calculations [23], a drawback is that it requires an *ansatz* for the symmetry of the density profile. In [23] we restricted ourselves to the investigation of planar structures formed at the planar surfaces. Indeed, it turned out that (neutral or hydrophilic) surfaces strongly stabilize the formation of bilayers consisting of two layers of oppositely oriented Janus particles. However, since the microscopic structure was assumed to be translationally invariant along the walls, it was impossible to detect, e.g., micelles.

Indeed, in a subsequent study of the *bulk* properties of the Janus model, this time on the basis of MD simulations [25], we found that micelles (of various sizes and shapes) are in fact the preferred structures for a broad range of densities and coupling strengths. One may therefore speculate that, at least in dilute systems and not too narrow surface separations, micelles should be observable again.

Against this background, the goal of the present MD study is twofold. First, by choosing a “representative” slit-pore confinement and some selected state points, we aim to explore the competition between interaction-induced micelle formation and surface-induced planar ordering (as suggested by the DFT). Second, to better understand the capabilities of the DFT approach we explicitly compare MD and DFT density profiles.

The remainder of this paper is organized as follows. The model and the method of investigation are briefly described in Section 2. In Section 3 we give a brief summary of our previous MD results for the bulk properties of the Janus particles [25]. Our results for confined systems are presented and discussed in Section 4. We close in Section 5 with a brief summary and outlook.

## 2. Model and Method

To describe the system of amphiphilic Janus particles, we employ a model originally introduced by Tarazona and coworkers [18]. In this picture, the particles are represented by spheres with an internal degree of freedom, that is, a unit vector **û***_i_* pointing from the hydrophobic to the hydrophilic hemisphere of each particle *i*. Following our former MD study of the bulk system [25], the pair interaction between two Janus particles then splits into a soft-sphere repulsion and an effective, solvent-mediated anisotropic pair interaction, that is

(1) $$\varphi (r_{ij},{\hat{u}}_{i},{\hat{u}}_{j})=\varphi^{\text{soft}}(r_{ij})+\varphi^{\text{I}}(r_{ij},{\hat{u}}_{i},{\hat{u}}_{j})$$

where **r***_ij_* = **r***_i_* *–***r***_j_* is the distance vector between the particles, and *r_ij_* = *|***r***_ij_* *|*. The soft sphere potential is defined as

(2) $$\varphi^{\text{soft}}(r_{ij})=4ɛ{\left(\sigma /r_{ij}\right)}^{12}$$

where *σ* is the diameter of the Janus particles. In our simulations we fix the strength of the repulsion, *ɛ*, such that *k*_B_*T/ɛ* = 1, with *k*_B_ and *T* being Boltzmann’s constant and temperature, respectively. The anisotropic pair interaction potential reads, to lowest order (see [18,25])

(3) $$\varphi^{\text{I}}(r_{ij},{\hat{u}}_{i},{\hat{u}}_{j})=\varphi_{1}(r_{ij})\;({\hat{u}}_{i}-{\hat{u}}_{j})\cdot {\hat{r}}_{ji}$$

where *φ*_1_ (*r_ij_*) is a Yukawa potential given as

(4) $$\varphi_{1}(r_{ij})=\frac{C}{r_{ij}}\text{exp }(-\lambda (r_{ij}-\sigma ))$$

The free parameters in Equation (4) are the coupling strength *C* and the inverse interaction range, *δ*. The latter is set to 3*σ^−^*^1^ in this study. The present model of amphiphilic Janus particles favors antiparallel side-by-side orientation, where the hydrophobic sides point towards one another. The opposite orientation (*i.e*., facing hydrophilic sides) is energetically most unfavorable, while parallel side-by-side and head-to-tail configurations are energetically neutral due to a vanishing anisotropic pair interaction (*cf.* Equation (3)). A more detailed discussion of the angle dependence and a comparison to other Janus models can be found in [23,25].

Spatial confinement is introduced by the presence of two planar, structureless, soft walls at the Cartesian positions *z*_wall_ = 0 and *z*_wall_ = *L*_z_. Each of these walls leads to a particle-wall potential of the form

(5) $$\varphi^{\text{softwall}}(z_{i})=\frac{4}{45}\rho_{\text{wall}}\pi {ɛ}_{\text{wall}}\frac{\sigma^{12}}{|z_{i}-z_{\text{wall}}{|}^{9}}$$

where we fix the wall density *ρ*_wall_ and wall coupling strength *ɛ*_wall_ such that *ρ*_wall_*σ*^3^*ɛ*_wall_ = *ɛ*. In the present study we consider the case *L*_z_ = 10*σ*. Also, we restrict ourselves to the discussion of neutral walls, thus focusing on the effect of confinement alone. In principle, however, it is straightforward to introduce surface potentials describing hydrophilic or hydrophobic walls; we have suggested corresponding potentials in our earlier DFT study [23] of confined Janus systems.

In the present work, calculations are carried out using equilibrium molecular dynamics (MD) simulations involving *N* = 1000 particles. The simulations are started by putting the particles on the sites of a square lattice, with random orientations and velocities, and zero angular momenta. The translational and rotational equations of motion are then integrated in combination with a Berendsen thermostat [26]. To check the equilibration in our simulations we consider the average value of the total energy as a function of time. In a fully equilibrated system, this function should become constant. For each thermodynamic state we perform one run. Within such a run, time averages over energies and related quantities are typically taken over a time interval of 3 million time steps with 6000 equidistant and uncorrelated measurements. The time averages concerning cluster distributions and correlation functions are taken over 2 to 3 million time steps. Further technical details of our MD simulations can be found in [25]. The thermodynamic state of the system is characterized by the reduced temperature T^*^ = *k*_B_*Tσ/C* and the reduced density ρ^*^ = *ρσ*^3^.

To analyze the structure formation in the slit-pore we calculate the usual number density profile, *ρ*(*z*) = *〈N_z_〉 /*(*A*Δ*z*) (with *A* being the box area parallel to the walls and Δ*z* being the thickness of the slice containing *N_z_* particles). In addition, we calculate an order parameter function characterizing the local “polarization”, that is

(6) $$h\;(z)=\langle \frac{\sum_{i=1}^{N_{z}}{\hat{u}}_{i}\cdot {\hat{e}}_{z}}{N_{z}}\rangle$$

where **ê***_z_* is a unit vector pointing along the *z*-axis. It follows from Equation (6) that *h* (*z*) can vary between *−*1 and 1, with *−*1 (+1) meaning that the hydrophilic (hydrophobic) sides of all particles point towards the left wall.

Furthermore, to investigate the lateral structure within layers of particles formed parallel to the walls, we calculate the in-plane radial distribution function defined as

(7) $$g_{2\text{D}}(r_{\left|\right|})=\frac{\langle N_{\text{layer}}\left(r_{\left|\right|},\Delta r_{\left|\right|}\right)\rangle }{\langle N_{\text{layer}}\rho_{\text{layer}}\rangle \pi \left({\left(r_{\left|\right|}+\frac{1}{2}\Delta r_{\left|\right|}\right)}^{2}-{\left(r_{\left|\right|}-\frac{1}{2}\Delta r_{\left|\right|}\right)}^{2}\right)}$$

In Equation (7)*N*_layer_ (*r_jj_,*Δ*r_jj_*) is the number of particles in a ring of width Δ*r_jj_* at a distance *r_jj_* within the layer considered. Further, *N*_layer_ is the total number of particles in the layer, and *ρ*_layer_ is the corresponding area density.

## 3. Background: Aggregation in the Bulk System

Before discussing the self-assembly in the slit-pore, we briefly summarize relevant results from our previous, extensive MD study [25] of the three-dimensional Janus-particle system. One main result from that study was an “aggregation line” T^*^_agg_(ρ^*^), which denotes (for each density ρ^*^) the temperature below which significant cluster formation occurs. Interestingly, we found no indication of a conventional *condensation* transition or coexistence between clustered phases (such as it has been predicted for another Janus model, see [16].

**Figure 1:**
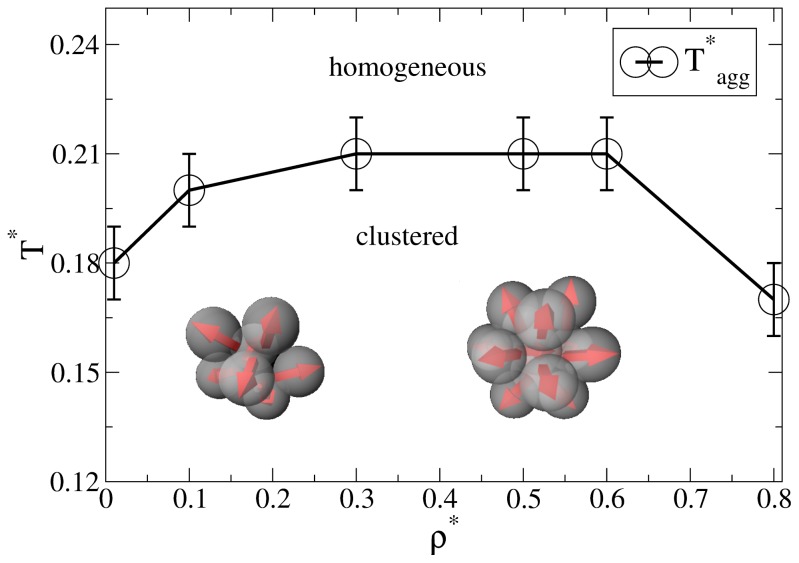
Aggregation line of the bulk system determined from the cluster size distribution. The line connecting the data points is a guide for the eye. The inserted sketches indicate typical cluster shapes at low and higher densities, respectively.

As discussed in [25], a convenient way to define the aggregation line is to monitor the cluster size distribution *N*_C_ (*s*), *i.e*., the number of clusters *N*_C_ of size *s*. For each temperature and density, this distribution is determined on the basis of a cluster search algorithm. At high temperatures, *N*_C_ (*s*) decays essentially exponentially, indicating a random distribution of cluster sizes. Upon lowering the temperature, however, one observes the emergence of a minimum at *s >* 1, indicating the existence of bound clusters [27]. The bulk aggregation line T^*^_agg_(ρ^*^) derived from that criterion is plotted in Figure 1. At low densities (ρ^*^ *≤* 0.3), the typical cluster sizes are between 5 and 10 [25]. Specifically, the Janus particles aggregate into spherical micelles where the hydrophilic sides of each particle is facing away from the cluster center (see the left sketch in Figure 1). For higher densities (0.3 < ρ^*^ *≤* 0.8), the cluster size distributions exhibits a pronounced peak at *s* = 13. As shown in [25] (via an analysis of correlation functions), these structures correspond to icosahedrons, *i.e*., close-packed aggregates which cannot be periodically continued to give a translationally ordered lattice [28,29]. Interestingly, the formation of these icosahedrons is accompanied by hindered translational and orientational dynamics, as reflected by various MD time correlation functions [25].

## 4. Confined Systems

In the following we study the behavior of the spatially confined Janus-particle system, focusing on state points below the bulk aggregation line (see Figure 1). Specifically, we consider confined systems, whose *average* density *ρ*_av_ = *L*_z_ *^−^*^1^ *∈*_0_*^L^*^_z_^ *dz ρ*(*z*) takes the values ρ^*^_av_ = 0.1 and ρ^*^_av_ = 0.65. We compare these systems to bulk systems at the same (bulk) density. It is well known that this way to “relate” bulk and confined systems is somewhat ambiguous since in the confined system, the regions close to each wall are effectively inaccessible to the particle’s centers of mass. The proper way to circumvent this problem is to work in the grand canonical ensemble, where the confined system can be uniquely related to the bulk by choosing the same *chemical potential* (rather than the same *ρ*_av_). However, to get a first insight into the impact of confinement, here we rather choose the simplified way described above. We also note that, for the (soft) particle-wall potential given in Equation (5), one could define an effective density via

(8) $$\rho_{\text{eff}}^{*}=\frac{\sigma^{3}}{L_{z}-\sigma }\int_{\frac{\sigma }{2}}^{L_{z}-\frac{\sigma }{2}}dz\rho \;(z)$$

This choice takes into account that the region inaccessible by the particles at each wall has (in total) approximately a thickness of one particle diameter. With this formula, the average densities ρ^*^_av_ = 0.1 and ρ^*^_av_ = 0.65 correspond to ρ^*^_eff_ = 0.111 and 0.722, respectively. In the subsequent two paragraphs we first present MD results for these two densities. Finally, we briefly compare the MD results to corresponding ones from our previous DFT study [23].

### 4.1. Low Densities

**Figure 2:**
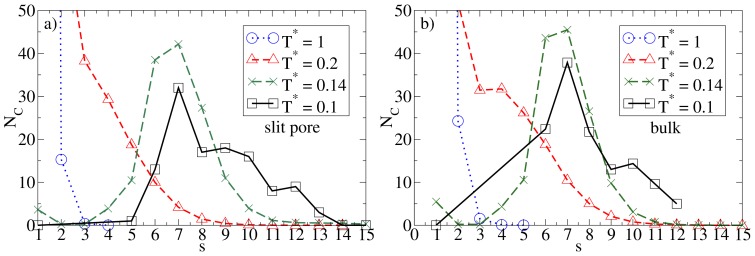
Cluster size distribution *N*_C_(*s*) (with *s* being the cluster size) and various temperatures for (**a**) the confined system at ρ^*^_av_ = 0.1 (ρ^*^_eff_ = 0.111); and (**b**) the bulk system at ρ^*^ = 0.1.

**Figure 3:**
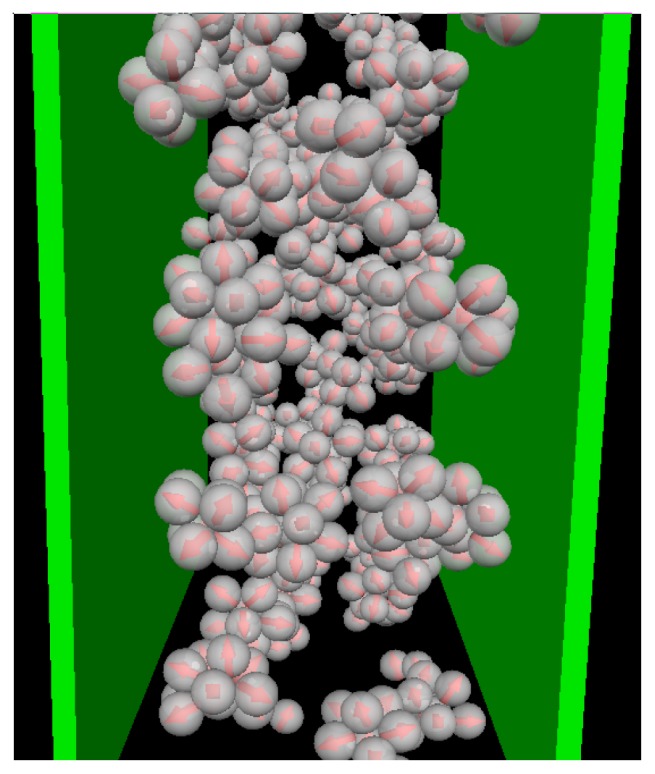
Snapshot from MD simulations at ρ^*^_av_ = 0.1 and T^*^ = 0.1.

Given the relatively large wall separation considered in the present study (*L*_z_ = 10*σ*), one would expect the confinement effects to be relatively weak as long as the average density is low. That this is indeed the case can be seen, e.g., from the cluster size distributions *N*_C_(*s*). Results for this quantity in the confined system and in the bulk are given in Figure 2, where we consider some characteristic temperatures. At T^*^ = 1.0, both systems are essentially homogeneous as indicated by the monotonic decay of *N*_C_(*s*). Bulk system starts to aggregate into spherical micelles at T^*^ *≈* 0.2 (see also Figure 1) as indicated by the appearance of a minimum in *N*_C_(*s*). This minimum is reproducible, as we have explicitly checked by performing several runs [25]. Interestingly, the corresponding function of the confined system is still monotonic at T^*^ = 0.2, indicating that the confinement somewhat *hinders* the micelle formation. At the substantially lower temperature T^*^ = 0.14, however, both functions clearly indicate the presence of (micellar) clusters of size 5 *–* 9, with an average of *〈N*_C_*〉 ≈* 7. Moreover, the main peak is of similar magnitude; this holds also for the even lower temperature T^*^ = 0.1. To illustrate the structure of this strongly coupled, confined system we present in Figure 3 a corresponding simulation snapshot. It is seen that the particles tend to aggregate into micellar clusters in the *middle* of the pore, rather than at the surfaces.

**Figure 4:**
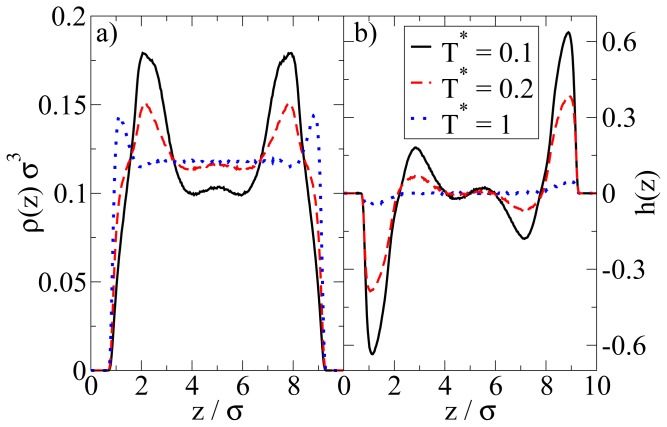
(**a**) Density profiles; and (**b**) polarization profiles at ρ^*^_av_ = 0.1 and three temperatures T^*^.

In Figure 4 we show the density and polarization profiles of the confined system at some temperatures above and below the aggregation line. As expected, the high temperature profiles (T^*^ = 1.0 ≫ T^*^_agg_) reflect an essentially homogeneous density distribution (expect directly at the walls) and the absence of any preferred alignment. A reduction of T^*^ results in a shift of the contact peaks of *ρ*(*z*) towards the center of the pore (see Figure 4a). This is a consequence of the aggregation also seen in the snapshot (*cf.* Figure 3): As soon as micelles have formed, their centers of mass are located at larger distances from the wall than what one would find in a non-aggregating system. At the same time, the development of negative (left) and positive (right) peaks in the order parameter profile plotted in Figure 4b reflects that the hydrophilic sides of the particles tend to be close to the walls. This is consistent with the preferred order within the micelles (where the hydrophilic sides of the particles point outwards). In other words, under the dilute conditions studied here, there seems to be no real competition between the structures favored by the particle interactions and those favored by the wall.

We note here that these findings (micelle formation) somehow contradict those in our previous DFT study [23]. There, the dominating structures even under dilute conditions are bilayers. We will come back to this point in Section 4.3.

### 4.2. High Densities

**Figure 5:**
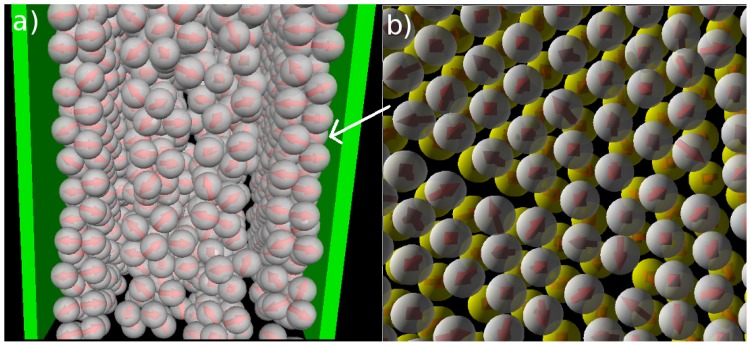
Snapshots from MD simulations involving soft walls (separation *L*_z_ = 10*σ*) at ρ^*^_av_ = 0.65 (ρ^*^_eff_ = 0.722) and T^*^ = 0.14. (**a**) Side view of the entire confined system; (**b**) top view onto one bilayer.

**Figure 6:**
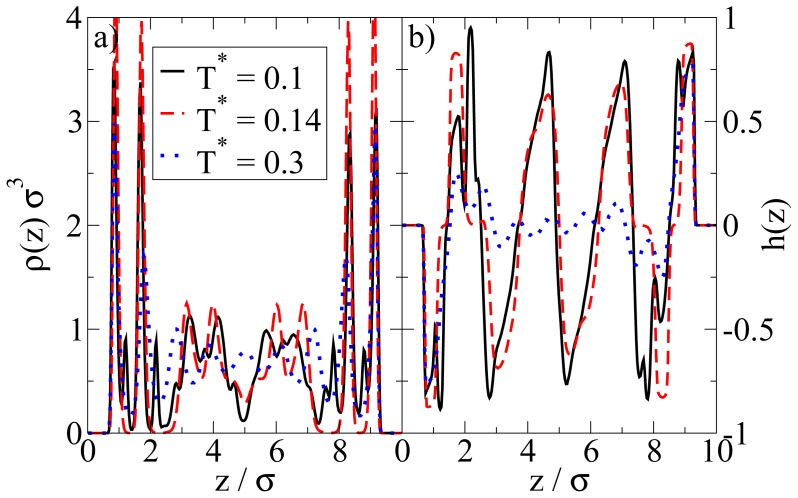
(**a**) Density profiles *ρ*(*z*); and (**b**) order parameter functions *h*(*z*) at ρ^*^_av_ = 0.65 for various temperatures T^*^.

We now consider confined systems at ρ^*^_av_ = 0.65 (ρ^*^_eff_ = 0.722). As expected, confinement effects at this high density are much more pronounced compared to the dilute case studied before. In particular, we find that there is a small range of temperatures (around T^*^ = 0.14), where the Janus particles form *bilayers* at the wall, contrary to the bulk system at the same density and temperature. In the latter, the preferred structure is the (spherical) icosahedron (see Figure 1). A snapshot of the confined system is shown in Figure 5a. From this snapshot one already sees that the bilayers are highly polarized in the sense that the particles in each single layer point outwards of the bilayer. These phenomena are also reflected by the density and polarization profiles, which are plotted (for various temperatures) in Figure 6. At the (somewhat higher) temperature T^*^ = 0.3, where the bilayers are not yet formed, the density profile (*cf.* Figure 6a) has the typical oscillatory shape reflecting layer formation of a dense fluid in a slit-pore confinement. The corresponding polarization profile, on the other hand, already indicates a preferential orientation of the Janus particles close to the walls (*cf.* Figure 6b). Both profiles significantly change when we now consider the case T^*^ = 0.14. Here, the formation of bilayers (see Figure 5a) leads to two sharp density peaks close to each wall. Further, we observe a clear depletion area at distances between the double layer and the pore center. Finally, the regular layered structure observed in the pore center at higher temperatures appears to be disturbed at T^*^ = 0.14. The corresponding polarization profile supports our former statement that the bilayers are highly polarized with the hydrophilic particle sides pointing outwards (*cf.* Figure 6b). One also sees from *h*(*z*) that the particles related to the two density peaks around the pore center are highly polarized. We interpret this behavior as a signal of the formation of spherical clusters, consistent with what is seen in Figure 5a.

**Figure 7:**
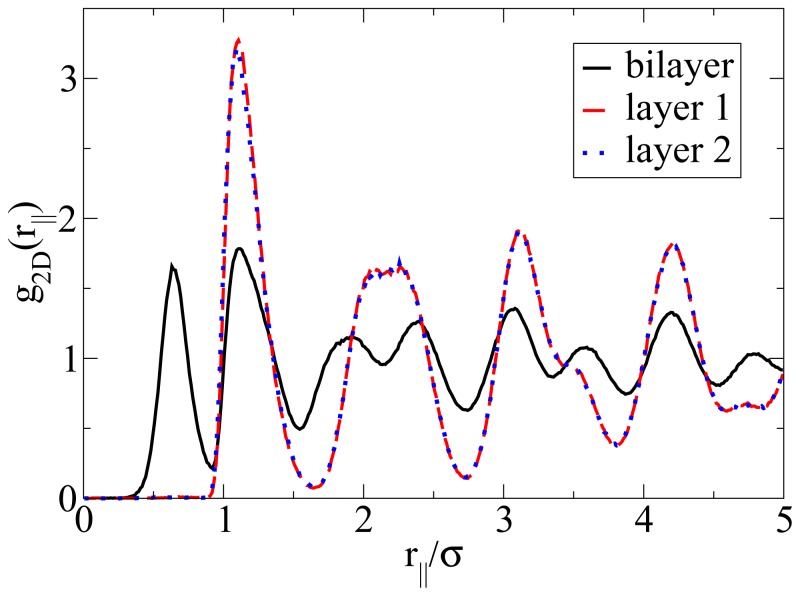
In-plane radial distribution function *g*_2D_(*r_jj_*) evaluated for the single layers and the complete bilayer at ρ^*^_av_ = 0.65 and T^*^ = 0.14.

Apart from the vertical ordering, it is also interesting to inspect the *lateral* structure within the bilayers formed at T^*^ = 0.14. Indeed, as shown in part (b) of the snapshot in Figure 5, the particles arrange into a highly ordered (yet not perfect) configuration with hexagonal-like symmetry. In this two-dimensional crystal-like structure, the positions of the particles in the two single layers are shifted relative to one another. These features are also reflected by the corresponding in-plane correlation functions, which are plotted as a function of the lateral distance in Figure 7. Specifically, we show the functions *g*_2D_(*r_jj_*) for each of the individual layers forming the bilayer, as well as for the complete bilayer. Considering first the two single-layer correlations, we find from Figure 7 that these are essentially indistinguishable. In other words, the internal structure within each layer is identical. To interpret the shape of these correlations, we note that the function *g*_2D_(*r_jj_*) of a *perfect* hexagonal lattice would display peaks at 1*σ*, $\sqrt{3}\sigma$ and 2*σ*. In the present, thermal system, we observe one peak at approximately 1.1*σ* and a broad maximum between approximately 2*σ* to 2.3*σ* (for each single layer). This “softening” (as compared to the perfect lattice) reflects the presence of thermal motion and lattice defects, which are also apparent from the snapshot in Figure 5b. To complete the discussion of the lateral structure, we note from Figure 7 that the *g*_2D_(*r_jj_*) evaluated for the entire bilayer clearly differs from that within each layer. This difference reflects the relative shift of the two layers along a direction parallel to the wall.

**Figure 8:**
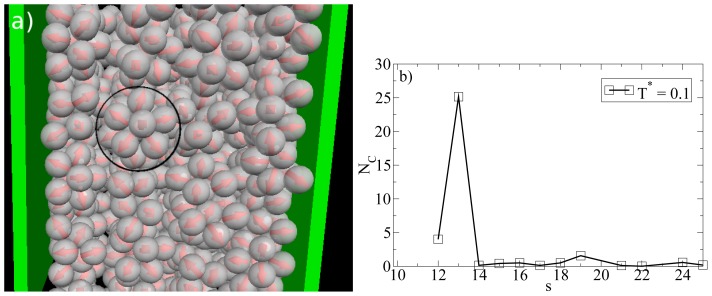
(**a**) MD simulation snapshot of the confined system (side view) at ρ^*^_av_ = 0.65 (ρ^*^_eff_ = 0.722) and T^*^ = 0.1. The black circle indicates an icosahedron; (**b**) Cluster size distribution *N*_C_(*s*) at ρ^*^_av_ = 0.65 and T^*^ = 0.1.

So far we have concentrated on the bilayer formation at T^*^ = 0.14. We stress again that these structures are purely surface-induced in the sense that the corresponding bulk system forms not planar, but rather spherical, specifically *icosahedron* structures. Interestingly, it turns out that this behavior is recovered also in the confined system, when we further lower the temperature (we recall in this context that our dimensionless temperature T^*^ measures the coupling strength of the anisotropic particle interactions). This change becomes apparent from Figure 8a, where we show a snapshot of the entire confined system at T^*^ = 0.1. Instead of bilayers (or any clearly defined layers at all), we find that the particles indeed arrange into icosahedrons (see circle in Figure 8a). Further, the density profile changes such that the peaks close to the walls become somewhat smaller and give rise to two additional peaks reflecting the different aggregation (*cf.* Figure 6a). We note that the density profile does not possess depletion areas at T^*^ = 0.1. An additional order parameter (*G*_cluster_^101^) [25] indicating the orientation of hydrophilic sides relatively to the cluster center shows a significantly smaller value than 1 at T^*^ = 0.14 and a value of 0.77 at T^*^ = 0.1. This means that aggregates at T^*^ = 0.1 are more spherical in average. We note that *G*_cluster_^101^ is even larger for a bulk system of icosahedrons, which leads to the assumption that micelles close to the wall are somewhat deformed with respect to an icosahedral local structure. The strong preference of this cluster type, which involves 13 particles, is also indicated by the corresponding cluster size distribution. The latter is shown in Figure 8b.

We conclude from this section that the surfaces are capable of influencing the self-assembly of the Janus particles only in a small window of reduced temperatures (around T^*^ = *k*_B_*Tσ/C ≈* 0.14). Indeed, when the particle-particle interactions become even stronger against the thermal energy (*C/k*_B_*Tσ ≥* 10), we observe “reentrant” bulk behavior, that is, formation of micelles.

### 4.3. Comparison to Density Functional Theory

As mentioned before, we have previously investigated the self-assembly at planar surfaces and in slit-pores via classical density functional theory [23]. Given that DFT is so far the only reliable theory to describe the structure of inhomogeneous fluids, it is thus of fundamental interest to check the DFT results against those from quasi-exact MD simulations. We note that a detailed comparison of the data is handicapped by several facts. First, within our DFT calculations, the particles are modeled as *hard* spheres, and similarly, the walls are hard as well. This choice enabled us to treat the contribution from the repulsive interactions to the excess free energy functional via Fundamental Measure Theory (FMT) [30,31]. which is known to be quasi-exact for pure hard spheres (the contribution from the anisotropic interactions, on the other hand, was treated by simple mean-field theory). Second, DFT calculations are performed in the grand canonical ensemble. Third, and perhaps most importantly, the DFT calculations are performed assuming translational invariance of the singlet density along the directions parallel to the walls. Thus, we could only detect formation of *planar* structures, but not of micelles or other non-planar objects. Given all these points, we limit ourselves here to a qualitative comparison of the *z*-dependent density profiles.

**Figure 9:**
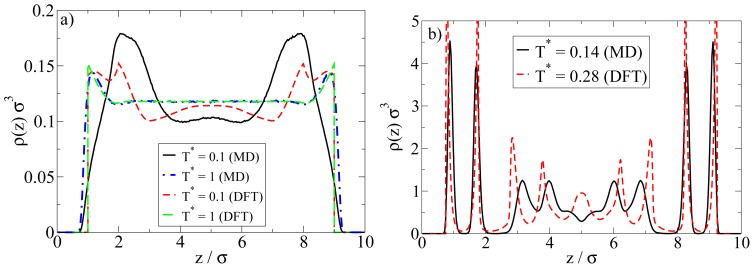
Comparison of DFT and MD density profiles at (**a**) ρ^*^_av_ = 0.1 (MD); and (**b**) ρ^*^_av_ = 0.65 (MD). For the DFT parameters, see main text.

Examples are plotted in Figure 9. We first consider the low-density situation (see Figure 9a). Specifically, the density chosen in the MD calculations is ρ^*^_av_ = 0.1 (as before) whereas in the DFT, ρ^*^_av_ = 0.107 (0.096) (corresponding to ρ^*^_bulk;DFT_ = 0.12 (0.1)) for T^*^ = 1.0 (0.1). We note in this context that, in order to approximately match the positions of the density peaks close to the walls, the DFT calculations have been performed with a wall separation of *L*_z_ = 9*σ* rather than 10*σ* as in the MD calculations.

At high temperatures above the aggregation line, such as T^*^ = 1.0, there is rather good agreement between MD and DFT calculations. Both methods predict an essentially homogeneous distribution of particles in the pore, accompanied by a nearly vanishing polarization profile (not shown). However, pronounced differences appear at low temperatures such as T^*^ = 0.1. Here, the density peak positions predicted by MD are shifted (relative to the high-temperature case) towards the center of the system, reflecting the formation of spherical clusters preferably in the middle of the pore (see also Figures 3 and 4 discussed in Section 4.1). Contrary to the MD profiles, the DFT profiles at T^*^ = 0.1 are characterized by a double peak close to each wall (located at *z* = 1.2*σ* and 2*σ*), possibly indicating the beginning of formation of a bilayer. We stress, however, that our DFT calculations are purely one-dimensional and thus do not *allow* for detection of a spherical clusters. In view of the MD results this is clearly a severe limitation of the DFT calculations when applied to low density Janus systems.

A comparison of the density profiles at a higher density is given in Figure 9b. The average densities chosen in the MD (DFT) calculations are ρ^*^_av_ = 0.65 (ρ^*^_av_ = 0.64). For each method, we focus on temperatures where bilayer formation occurs. Indeed, given that our DFT involves a mean-field approximation for the anisotropic interactions and that we allow for planar structures alone, it is not surprising that this approach predicts bilayers already at much larger reduced temperatures than the MD. Specifically, the DFT results in Figure 9b pertain to T^*^ = 0.28, whereas those from MD correspond to T^*^ = 0.14 (see Figure 5 for a snapshot).

Inspection of Figure 9b shows that, within the *z*-range related to the bilayers, the DFT profile is sharper, but otherwise similar to that from MD. Moreover, the DFT predicts some kind of depletion zone between the bilayer and the structures in the pore center, which is again consistent (on a qualitative level) with the MD data. Larger differences are seen in the region around the pore center. Here, the DFT predicts further (yet less pronounced) bilayers, whereas the MD data rather reflect more complex spherical structures (see discussion in Section 4.2).

## 5. Conclusions

The main purpose of this paper was to present MD simulation results for the self-assembly of amphiphilic Janus particles in a slit pore. We focused on the case of a relatively wide pore, characterized by a wall separation of ten (effectively nine) particle diameters, and neutral walls. At small average densities, the impact of the surfaces is found be quite minor. Indeed, we observe essentially the same structures, that is, spherical micelles, as in the bulk system at the same density and temperature (*i.e*., coupling strength); even the cluster size distributions at low temperatures have similar shape. The micelles in the confined system form preferably in the pore center, whereas the regions around the (purely repulsive) surfaces turn out to be rather depleted. In other words, the surfaces somewhat perturb the micelle formation. This could explain why, upon cooling the system from high temperatures, the *onset* of micelle formation (as detected from the cluster size distribution) occurs at somewhat lower temperatures than in the bulk.

Contrary to the low-density case, confined Janus-particles systems at high average densities can display structures strongly different from their bulk counterpart. Indeed we found that, within a small range of reduced temperatures, the surfaces stabilize bilayer structures characterized by a high degree of polarization relative to the walls. Such structures are absent in a bulk system at the same temperatures and (average) density. At even lower temperatures, however, the anisotropic particle-particle interactions become again dominant, and one observes formation of icosahedral micelles, consistent with the bulk. This reentrant behavior indicates that dense, confined Janus-particle systems are subject to a strong competition between planar structures preferred by the surfaces and non-planar (micellar) structures induced by particle-particle interactions.

In the last part of the paper, we have compared MD density profiles of the confined systems to density profiles obtained by classical DFT [23]. Since the DFT calculations employed here only allow for *one-dimensional* inhomogeneities perpendicular to the surfaces, it is impossible to detect formation of micelles (or any non-planar structures) by this method. Thus, it is not surprising to see that the DFT essentially fails in the description of dilute, confined systems where micelles are dominant. On the other hand, the bilayer structures found at higher densities are described fairly well, at least on a qualitative level.

Clearly, the present MD study has rather exploratory character in the sense that we considered only one wall separation, and only one type of surfaces here. Regarding the wall separation, it would be interesting to explore, on the one hand, the case of larger *L*_z_ where both bilayers at the walls and micelles in between can be formed. On the other hand, for wall separations of the order of the particle diameter, our previous DFT study [23] suggests interesting frustration effects. Regarding the type of surface, it would be very interesting to consider the case of additional surface fields, such as surfaces with hydrophilic or hydrophobic coatings [20]. Indeed, based on our previous DFT study of dense suspensions at such surfaces [23], we would expect that hydrophilic surfaces strongly stabilize bilayer formation, whereas hydrophobic walls could enforce totally different structures. Another open question concerns the impact of *curved* surfaces. An example is the inner surfaces of the (typically cylindrical) pores of an ordered porous material. This question has already also been investigated in the context of the self-assembly of amphiphilic molecules [32,33]. We expect a similar competition between surface- and interaction-induced ordering for Janus particles at curved surfaces. Finally, it would be very interesting to investigate the role of the size of the hydrophilic versus that of the hydrophobic part of the Janus particles for their self-assembly in confinement. That these particle-related details could be important has already been suggested in a previous theoretical investigation for bulk systems, yet on the basis of a different model [15].
